# Whole-exome sequencing data of suicide victims who had suffered from major depressive disorder

**DOI:** 10.1038/sdata.2019.10

**Published:** 2019-02-05

**Authors:** Dóra Tombácz, Zoltán Maróti, Tibor Kalmár, Miklós Palkovits, Michael Snyder, Zsolt Boldogkői

**Affiliations:** 1 Department of Medical Biology, Faculty of Medicine, University of Szeged, Somogyi B. u. 4., Szeged, H-6720, Hungary; 2 Department of Paediatrics, Faculty of Medicine, University of Szeged, Korányi fasor 14-15., Szeged, H-6720, Hungary; 3 Neuromorphological and Neuroendocrine Research Laboratory, Department of Anatomy, Histology and Embryology, Semmelweis University, Üllői u. 26., Budapest, H-1085, Hungary; 4 Department of Genetics, School of Medicine, Stanford University, 300 Pasteur Dr., Stanford, CA, 94305-5120, USA

**Keywords:** Molecular neuroscience, Next-generation sequencing, Medical genomics

## Abstract

Suicide is one of the leading causes of mortality worldwide; it causes the death of more than one million patients each year. Suicide is a complex, multifactorial phenotype with environmental and genetic factors contributing to the risk of the forthcoming suicide. These factors first generally lead to mental disorders, such as depression, schizophrenia and bipolar disorder, which then become the direct cause of suicide. Here we present a high quality dataset (including processed BAM and VCF files) gained from the high-throughput whole-exome Illumina sequencing of 23 suicide victims – all of whom had suffered from major depressive disorder - and 21 control patients to a depth of at least 40-fold coverage in both cohorts. We identified ~130,000 variants per sample and altogether 442,270 unique variants in the cohort of 44 samples. To our best knowledge, this is the first whole-exome sequencing dataset from suicide victims. We expect that this dataset provides useful information for genomic studies of suicide and depression, and also for the analysis of the Hungarian population.

## Background & Summary

Suicide is the 10th leading cause of mortality in the world^1,2^. It is a complex, multifactorial behavioural condition in which proximal and distant risk factors, such as mental disorders, genetic and epigenetic factors, social, cultural and economic environment, gender, age, personality, eating disorders, as well as drug or alcohol abuse^3–7^ play important roles. The presence of early child abuse has also been described as a contributing factor to suicidal behaviour^8^. Studies have repeatedly shown that suicide is most common among patients with mood disorders, such as major depressive disorder and bipolar disorder^9^.

Whole-exome sequencing (WES) is a cost-effective and powerful tool for the analysis of complex and rare genetic diseases^10^. WES technique allows a base-pair comparison of exomes and consequently the examination of rare genetic variants, which may play a role in suicide. Alterations in copy number variations (CNVs) and short-tandem repeats (STRs), as well as the viral infections can be also analysed by WES.

We performed high-depth, paired-end, short-read WES on 23 suicide victims suffered from major depressive disorder (MDD; this information is available in EGA sample metadata) and 21 control patients who died from other diseases (EGA sample metadata). Harvesting of brain tissues was approved by the local ethics committee^11^. Informed consents from the next kin have not been obtained from the victims because the local regulations did not require consent for autopsy. All data were handled anonymously. Both of the cohorts were of Hungarian ethnicity. The Hungarian population is known to have had a very high annual suicide rate in the previous century^12,13^. The DNA samples derived from post-mortem brains were sequenced using the Agilent SureSelectXT Human All Exon V5 + UTRs kit and Illumina HiSeq 2000 sequencing platform resulting in an average of 117 million paired-end reads.

We obtained very high coverage per base position in both cohorts; in the suicide cohort 95.4–97.5% (quartiles) of target regions had higher than 20-fold and 82.8–90.5% of target regions had higher than 40-fold coverage. In the Hungarian control cohort these values were 96.3–97.5% for 20-fold and 81.6–91.6% for 40-fold coverage, respectively. We called on average ~130,000 variants per sample. Altogether we observed 442,270 unique variants in the control and suicide cohorts. Furthermore, we determined a total of 320,976 single nucleotide variants (SNVs) and 47,313 insertions/deletions (indels).

The library preparation kit used in this study, as well as the obtained very high coverage of sequencing reads that we obtained enabled us to capture information not only about the exons but also about the 5′- and 3′-UTRs, the promoters to a certain length, and even about off-target sequences, such as introns, intergenic regions and infecting viruses. Our dataset had 286,754 high coverage regions (exceeding 20-fold coverage) in all samples that can be suitable for downstream analysis of CNVs.

**Figure 1:**
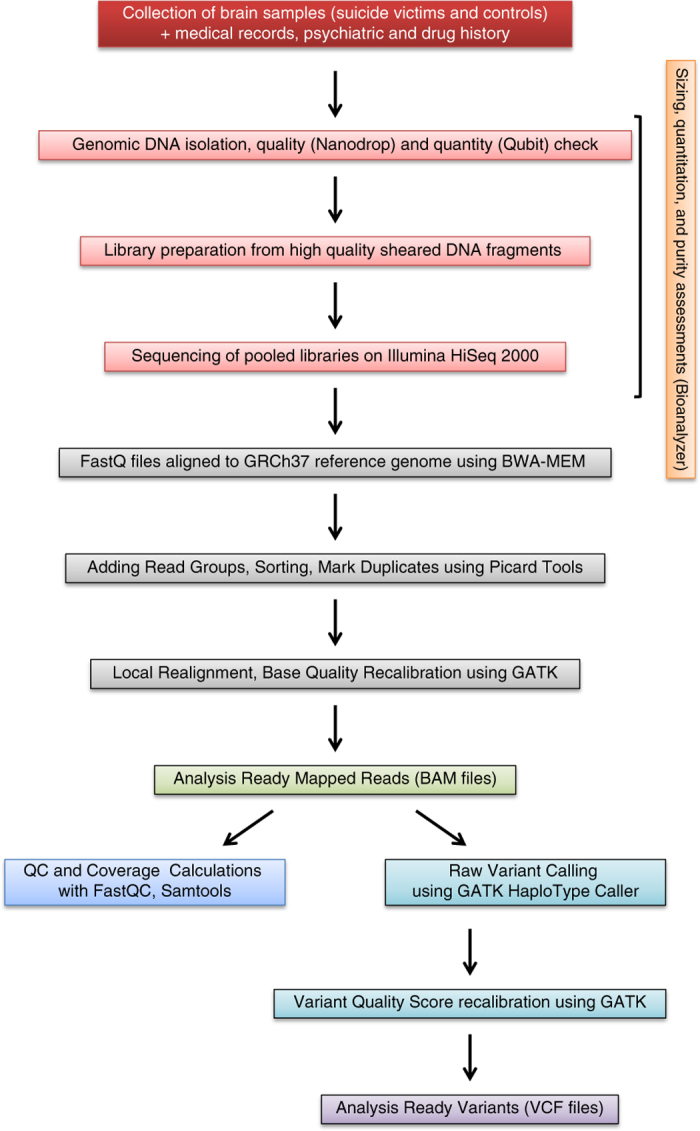
Data flow diagram shows the detailed overview of the study design, wet lab experiments and bioinformatics pipelines.

Here we describe the sample collection methods, the library preparation and sequencing method, the currently available data records, and technical validations for our dataset. A schematic overview of this study, including the experimental procedure and the bioinformatics workflow, is also presented (Fig. 1).

To our knowledge, this dataset is the first individual-level, open-access WES data release targeted to suicide. The dataset described here has been thoroughly analysed in our related manuscript^6^, where it has been shown that the TGF-β signalling pathway and three voltage-gated calcium ion channels may play an important role in the pathogenesis of suicidal behaviour. Our analysis proposed that suicide may be an umbrella-disease like disorder.

Here we provide the processed BAM files, as well as the processed Variant Call format (VCF) files for each of the samples containing the analysis ready variants of GATK HaplotypeCaller pipeline that allows for the testing of different variant filtration strategies that we had used in our original manuscript or for hypothesis testing of candidate genes (covered by SureSelect V5 All Exon plus UTR kit) without tedious bioinformatic processing.

Our dataset provided here in this manuscript can be re-used to investigate suicide and MDD. These data can be a valuable resource for investigating genetic variants, genes and signalling pathways to identify novel factors related to these disorders, and may provide novel information for the investigation of the Hungarian population or in general for studies of genetic polymorphisms of human population.

## Methods

The methods described here are an expanded version of those described in our recently published, related publication^6^.

### Medical history of the suicide victims

This study is based on whole-genome sequence data from occipital, cerebellar and somatomotor cortex regions of the brains of 23 suicide victims (15 males and 8 females) and 21 control patients (14 males and 7 females) who died suddenly from other causes (this information is available in EGA sample metadata). Samples were obtained by autopsy at the Department of Forensic Medicine of the Semmelweis University Medical School. For all of the suicide victims, a psychiatric diagnosis of major depression was on record. The diagnoses had been made by experienced psychiatrists on the basis of Diagnostic and Statistical Manual of Mental Disorders Fourth Edition (DSM-IV). Patients with a history of schizophrenia, epilepsy and other disorders were excluded from the study. The medical, psychiatric, and drug history of the suicide victims was obtained by psychological autopsy, which included interviews with the attending physician and with family members, as well as data obtained from medical records. The participants included in this study had no history of drug or alcohol abuse, and had not used antidepressant medication for at least two months prior to death. This information was confirmed by toxicology tests on blood samples. Suicide victims died by hanging (n = 16), drug overdose (n = 6), or jumping from height (n = 1). Causes of death in control subjects are presented in EGA sample metadata. Harvesting of tissues was approved by the local ethics committee.

### Tissue collection, dissection, and storage

Brains were obtained 1 to 10 h after death (this information is available in EGA sample metadata). After removal from the skull, the brains were cut in six major pieces (four cortical lobes, basal ganglia – diencephalon, and lower brain stem – cerebellum), rapidly frozen on dry ice, and stored at −70 °C until dissection (2 days to 2 months). At the time of dissection, the brain samples were sliced into 1 mm-thick coronal sections at a temperature of 0 to 10 °C. The cortical areas were cut out of the sections by a fine microdissecting (Graefe’s) knife. In all cases, cortical samples were always taken from the right hemisphere. The samples were stored in airtight containers or plastic tubes at −70 °C until further use.

### DNA purification

Genomic DNA (gDNA) samples were isolated from the occipital, cerebellar or somatomotor cortex regions (Table 1 (available online only)) using the DNeasy Blood and Tissue Kit (Qiagen) according to the manufacturer’s recommendations for the spin-column protocol, using 30 mg staring tissue material from each sample. In short, tissue samples were cut into small pieces, and then lysis buffer (provided by the kit) and proteinase K were added. Lysis reactions were carried out at 56°C until complete lysis was obtained. DNeasy Mini spin columns (kit’s component) were used for the isolation of gDNA from the lysate. Elution was carried out twice to a final volume of 100 μl per elution.

### Quality and quantity check of DNA

The quality of the DNA was checked prior to downstream analysis by NanoDrop (Thermo Fischer Scientific) measurement. High-quality samples with an OD 260/280 ratio ranging from 1.8 to 2.0 were subjected to the next step. Qubit 2.0 fluorometer (Invitrogene) was used for genomic DNA quantification before library preparation (Table 1 (available online only)).

### Whole-exome sequencing

**Figure 2:**
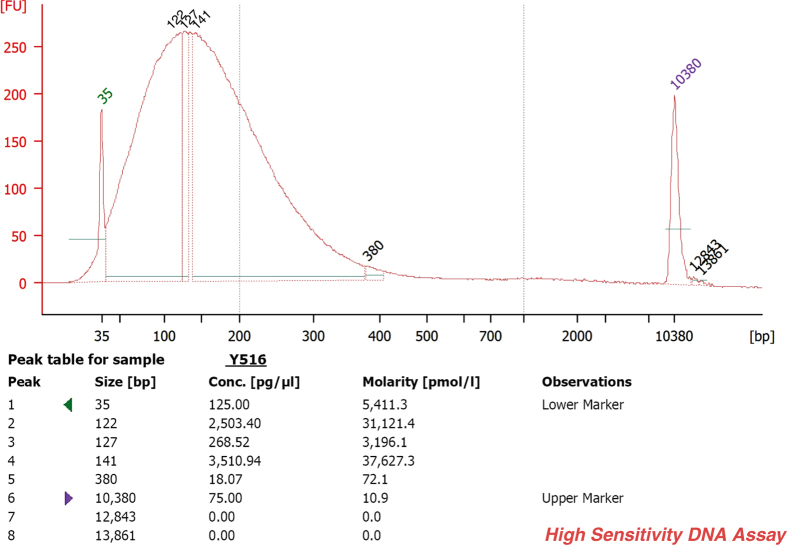
Bioanalyzer (BA) electropherograms of a representative sample (# Y516) at the first step in the library preparation workflow. The figure shows a representative example of the DNA fragment size distribution after sonication. The fragment size peak of this sample is 141 bps.

**Figure 3:**
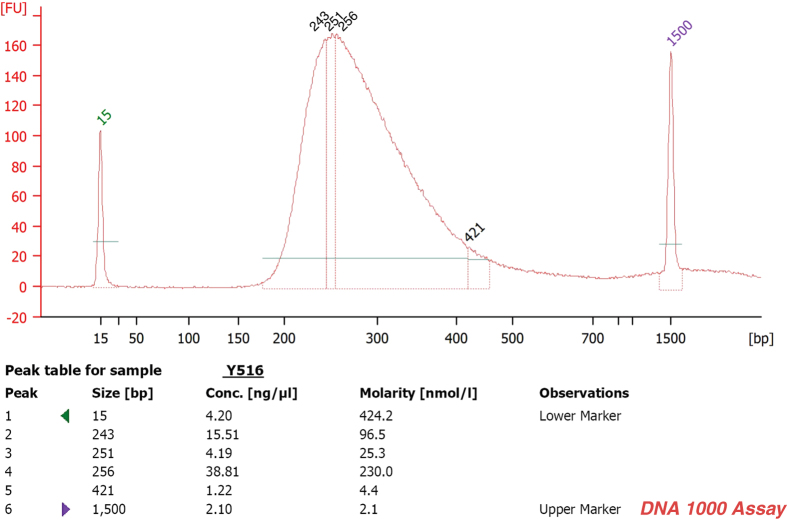
A representative sample peak is shown in this figure, which illustrates the quality of the amplified library. This electropherogram shows a distribution with a DNA fragment size peak of 256 bps for sample Y516.

**Figure 4:**
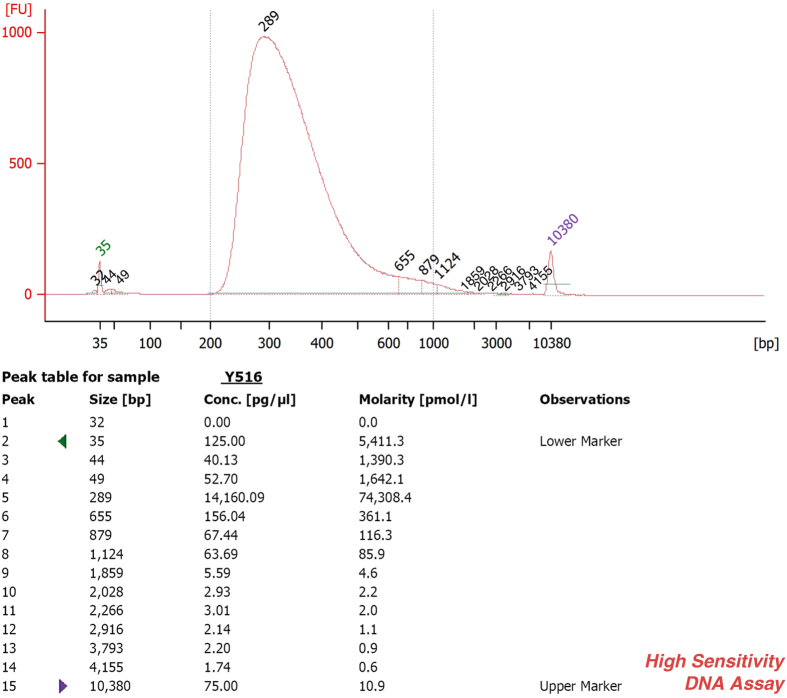
This figure represents the post-capture quality analysis of the PCR-amplified indexed library from the sample Y516. The size peak of this sample was 289 bps, while the average size was 351 bps.

Paired-end, indexed libraries for Illumina sequencing were prepared from the DNAs of the 23 suicide victims and the 21 controls using post-mortem brain cortex tissues as a source. Whole-exome sequencing was carried out as previously described^14^ with slight modifications. In short, 200 ng from each of the qualified gDNA samples in 100 μl volume were sheared into fragments of approximately 200–450 bps in Covaris microTUBEs (Covaris microTUBE AFA Fiber Pre-Slit Snap-Cap 6 × 16 mm) with Covaris s220 high-performance ultrasonicator. The quality of the DNA was checked prior to downstream analysis, using the Agilent Bioanalyser 2100 (Agilent Technologies) and the High Sensitivity DNA Analysis Kit (Agilent Technologies; Fig. 2). The end-repair, A-tailing (3′ adenylation) and ligation of the paired-end adaptors steps were executed with the Agilent SureSelect Human All Exon V5 + UTRs kit (Agilent Technologies) as was described in the following manual: SureSelect^XT^ Target Enrichment System for Illumina Paired-End Sequencing Library Illumina HiSeq and MiSeq Multiplexed Sequencing Platforms Protocol version 1.6 (SureSelect^XT^). Amplification of the adaptor-ligated libraries was performed using the Herculase II Fusion DNA polymerase (Agilent Technologies) following instructions as set forth in the above mentioned manual. Purification after the enzymatic reactions was carried out by using Agencourt AMPure XP beads (Beckman Coulter). Quality and quantity of the libraries were determined by 2100 Bioanalyser instrument and DNA 1000 Assay (Agilent Technologies; Fig. 3). DNA library samples were hybridized with target-specific Capture Library using the SureSelect^XT^ Reagent Kit, HSQ for the HiSeq platform (Agilent Technologies) based on the SureSelect^XT^ protocol. Incubation of the hybridization mixture was carried out at 65 °C for 24 h. After hybridization, the targeted molecules were captured on streptavidin-coated magnetic beads. The SureSelect enriched libraries were amplified by PCR with 6-bp indexing primers (Table 2 (available online only)) following the SureSelect^XT^ manual’s post-capture PCR recommendations using 12 cycles. The amplified libraries were purified using AMPure XP beads. The quality and quantity of the indexed libraries were checked by Agilent Bioanalyser and High Sensitivity DNA Assay (Fig. 4, Table 3). The quantities were also measured by Qubit 2.0 (Table 3). Two or three indexed libraries were pooled (Tables 2 (available online only) and 3) and the pooled samples were sequenced in one lane of a HiSeq 2000 flow cell, generating 101 bp paired-end reads.

### Sequence QC

Illumina HiSeq paired-end BCL files were converted to FASTQ files by the standard Illumina protocol (bcl2fastq) in order to remove the adapters, the known Illumina artefacts.

### Read Mapping, BAM file post processing

The trimmed, adapter removed FASTQ files were mapped to the GRCh37 reference genome by the Burrows-Wheeler Aligner (version 0.7.9a-r786) with the BWA MEM paired-end mapping algorithm using the following parameters: -M -w 150 -d 80 -c 2000. This mapper is generally recommended for high-quality queries as it is fast, and it allows gapped alignments which are essential for the accurate identification of SNV and insertion/deletions (indels; http://genestack-user-tutorials.readthedocs.io/tutorials/WES_data_analysis/index.html). Duplicates were marked by the Picard tools (version 1.113) MarkDuplicates algorithm. The raw BAM files (Data Citation 1) were realigned and base quality recalibrated by Genome Analysis Toolkit (GATK version: 3.3-0-g37228af).

### Sequence QC

FastQC (version v0.11.2) was used to generate quality reports (Data Citation 2) using the aligned and base quality recalibrated BAM files.

### Variant calling

The variants were called by the GATK HaplotypeCaller (version: 3.3-0-g37228af) algorithm using the GVCF mode cohort analysis according to the Best Practice Guidelines^15–17^. Raw Variants were recalibrated by GATK VariantRecalibrator according to the best practice recommendations using the HapMap, Omni, dbSNP, 1000 G, Mills datasets for training included in the GATK GRCh37 bundle resulting analysis ready variants (Data Citation 2).

## Data records

The raw FASTQ files, the GRCh37 aligned BAM files, and the variants in VCF 4.1 format associated with the samples analysed in this study are available on request at EGA (Data Citation 1). We also deposited the QC metric files of Picard MarkDuplicate, and QC reports of FastQC of the analysis ready Bam files at Figshare (Data Citation 2) and in Supplementary Table 1.

## Technical validation

### Quantitation of the purified DNA samples

The isolated DNA samples were quantified by Qubit (Life Technologies) fluorometer using Qubit dsDNA HS (High Sensitivity) Assay Kit, which is highly selective for double-stranded DNA over RNA and is designed to be accurate for initial sample concentration ranging from 10 pg/μl to 100 ng/μl. DNA samples were diluted to 4 ng/μl with 1X Low TE Buffer. NanoDrop (Thermo Fischer Scientific) measurements were also performed to assess quantity and quality of DNA, 260:280 and 260:230 ratios greater to 1.8 were accepted.

### Quality control of the sheared DNA samples

The quality of the sheared DNA samples (200 ng of each) were checked prior to downstream analysis, using the Agilent Bioanalyser 2100 (Agilent Technologies), and High Sensitivity DNA chip and reagent kit. The electropherogram showed a DNA fragment size peak (for each of the samples) at around 150 bps (Fig. 2).

### Quality check of the amplified samples

Agilent Bioanalyser 2100 (Agilent Technologies) and DNA 1000 Assay were used for the quality and quantity control of the libraries after PCR. The sample fragments sizes were between 225 and 275 bps (Fig. 3).

### Assessment of the quantity and quality of the indexed library DNAs

The quality of the amplified, indexed libraries were determined before multiplexing using the Agilent Bioanalyser 2100 (Agilent Technologies) and High Sensitivity DNA Assay (Table 3). The peak of the DNA fragment size was between 200–450 bps for each of the samples with an average of 355 bps (Fig. 4, Table 3).

### Technical replicates

Two independent technical replicate libraries were prepared and sequenced from the same patients (three suicide victims and two controls; Table 4).

### Quality control of raw reads

Illumina BCL files were converted to FASTQ files by the standard Illumina protocol to remove low-quality reads and adaptors.

### Quality control of mapped reads

We used Picard tools MarkDuplicate algorithm to identify PCR or optical duplicates. According to MarkDuplicates statistics, we had very low percentages of duplicated reads (on average 0.624%) in our cohort. Genome Analysis Toolkit (GATK version 3.3) BaseRecalibrator base quality score recalibration tool was used to generate the final quality recalibrated BAM files for downstream analysis and variant calling according to best practices^15–17^. To check the quality of our mapped reads we used FastQC to generate quality reports of base quality score recalibrated BAM files. According to the FastQC reports we had no adapter contamination or overrepresented sequences indicating contamination in any of the analysed sequences. Furthermore, the majority of sequences were of high quality (average Phred quality score >34) and even the last two base pairs of the sequences had an average Phred quality score >30. On average 99.9% of reads were mapped, 99% were properly paired, and we had less than 0.1% singletons. The average read counts in the cohort were 117 013 694.9 while the average insert sizes were in the range of 157.7–190.0 base pairs (Table 5 (available online only)). According to the main quality indicators of our dataset all data files fall within acceptable parameters as shown in Table 5 (available online only).

### Variant calling

The identification of different genomic variants including SNVs, indels, multiple nucleotide variants (MNVs), Genome Analysis Toolkit (GATK version 3.3) HaplotypeCaller algorithm was used with the GVCF mode cohort analysis according best practice^15–17^. HaplotypeCaller was run with the “-stand_emit_conf 10 -stand_call_conf 30” parameters. Variants were recalibrated by GATK VariantRecalibrator according to the best practice recommendations using the HapMap, Omni, dbSNP, 1000 G, Mills datasets for training included in the GATK GRCh37 bundle. We used 100.0, 99.9, 99.0, 90.0 tranches for variant recalibration and the 99.0 truth sensitivity level was used at ApplyRecalibration algorithm to identify QC PASS-ed variants.

## Usage Notes

Ethical approval for the study was obtained from the following institutional review boards:

The brain samples were collected by the Human Brain Tissue Bank (HBTB; Semmelweis University, Budapest). The activity of the HBTB has been authorized by the Committee of Science and Research Ethic of the Ministry of Health Hungary (ETT TUKEB: 189/KO/02.6008/2002/ETT) and the Semmelweis University Regional Committee of Science and Research Ethic (No. 32/1992/TUKEB) to remove human brain tissue samples, collect, store and use them for research.

The HBTB is a member of the BrainNet Europe II. consortium. The activity of the HBTB is in accordance with the ethic and safety rules and the scientific requirements of the consortium.

Human brain microdissection procedures were approved by the Regional and Institutional Committee of Science and Research Ethics of Scientific Council of Health (the number of the ethical license: 34/2002/TUKEB-13716/2013/EHR, Budapest, Hungary) and the Code of Ethics of the World Medical Association (Declaration of Helsinki).

Conducting genetic testing on tissue samples and then sending them abroad has been authorized by the Semmelweis University Regional Committee of Science and Research Ethics (No. 34/2002/TUKEB).

The Institutional Review Board (IRB) at Stanford has made the following determination about the activity of the study, titled “*Exome sequencing analysis of suicidal behavior using high-throughput DNA sequencing*”, based on Office for Human Research Protections (OHRP) and Food and Drug Administration (FDA) regulations and guidance: this project does not require submission to the Stanford IRB, because the study does not involve human subjects, it is not about living individuals (NOT-H3 1/1; November 26, 2014).

### The following explains how to access the dataset provided in this manuscript

The data is made available through the European Genome-phenome Archive (EGA). Search for the Data Access Committee (DAC) ID of our study (EGAD00001004184) at the homepage of EGA (https://ega-archive.org/). This will show detailed information on our dataset, data providers, DAC and any other related documentation. You can also find information about who to contact about access to this dataset. Access to data will be allowed to qualified researchers for appropriate health related studies.

A DAC group is responsible for the access of the datasets deposited in EGA.

Request for data access will be referred directly to our Data Access Committee: https://ega-archive.org/datasets/EGAD00001004184.

Data access decisions can be passed to the EGA in two ways: 1) By emailing helpdesk@ega-archive.org with the email address of each applicant and confirmation of the dataset/s to provide access. The EGA will then create an EGA account with the relevant access permissions. 2) By using the EGA DAC Admin tools (available to DAC’s dealing with more than 5 data access applications/month).

If you need to request access to this data set, please contact:

***DAC for Hungarian human exome team*** (Department of Medical Biology, University of Szeged, Hungary)

Contact person: Dr. Zsolt Boldogkői

Email: boldogkoi.zsolt@med.u-szeged.hu

Applicants will be asked to complete the Data Access Agreement (DAA) (including a brief summary of the proposal, proposed usage of the dataset, the storage of data, so the DAC can determine if the planned usage falls within the consents) and to agree to the terms and conditions of the DAA. The DAA must be signed by the applicant and the relevant Head of Department, or equivalent. If applications include a named collaborator then the collaborator’s Institution must sign and submit a separate DAA. A template DAA can be found on the EGA website: https://www.ebi.ac.uk/ega/sites/ebi.ac.uk.ega/files/documents/Example%20DAA.doc.

## Additional information

**How to cite this article**: Tombácz, D. *et al*. Whole-Exome Sequencing Data of Suicide Victims who had Suffered from Major Depressive Disorder. *Sci. Data*. 6:190010 https://doi.org/10.1038/sdata.2019.10 (2019).

**Publisher’s note**: Springer Nature remains neutral with regard to jurisdictional claims in published maps and institutional affiliations.

## Supplementary Material



Supplementary Table 1

## Figures and Tables

**Table 1:** This table summarizes the brain tissue regions that were used for preparation of genomic DNA, and the final concentrations (ng/ul) of the gDNA samples.

SAMPLE	BRAIN REGION	Cc
Y919	occipital cortex	14.5
Y166	occipital cortex	29.8
Y645	occipital cortex	15.2
BI269	occipital cortex	8.39
Y558	occipital cortex	7.73
Y591	occipital cortex	5.08
Y520	occipital cortex	15.3
Y440	occipital cortex	15.4
Y426	occipital cortex	5.78
Y787	occipital cortex	2.1
Y988	occipital cortex	2.24
Y482	occipital cortex	20.9
BIB82	occipital cortex	1.8
BI370	occipital cortex	6.08
YA205	occipital cortex	15.3
Y640	occipital cortex	10
BI919	occipital cortex	21.6
YA626	occipital cortex	11.9
YA29	occipital cortex	5.13
YB479	occipital cortex	2.23
YB330	occipital cortex	7.18
Y928	occipital cortex	6.68
Y974	occipital cortex	4.38
Y78	occipital cortex	14.5
YA762	occipital cortex	2.55
Y803	cerebellar cortex	18.9
Y915	cerebellar cortex	18.6
A438	cerebellar cortex	5.14
Y880	cerebellar cortex	10.6
YA243	cerebellar cortex	6.42
Y967	cerebellar cortex	11.8
Y726	cerebellar cortex	20.3
B168	cerebellar cortex	6.46
Y872	cerebellar cortex	35.5
Y64	cerebellar cortex	6.1
Y532	cerebellar cortex	4.91
Y375	cerebellar cortex	2.44
Y331	cerebellar cortex	2.25
Y316	cerebellar cortex	2.51
Y724	cerebellar cortex	16.1
Y292	cerebellar cortex	11.8
Y393	cerebellar cortex	7.23
Y421	cerebellar cortex	7.42
Y516	cerebellar cortex	8.77
Br969	somatomotor cortex	33.2
Br708	somatomotor cortex	6.11
Br333	somatomotor cortex	31.3
Br857	somatomotor cortex	21
BrA206	somatomotor cortex	17.2

**Table 2:** This table shows the samples, which were mixed and sequenced together.

RUN	SAMPLE	TYPE	BRAIN REGION	INDEX
1	Y919	suicide	occipital cortex	6
	Y166	suicide	occipital cortex	12
	Y645	suicide	occipital cortex	4
2	Y375	suicide	cerebellar cortex	12
	Y316	suicide	cerebellar cortex	6
	Y393	suicide	cerebellar cortex	4
3	Br333	suicide	somatomotor cortex	6
	Br857	suicide	somatomotor cortex	12
	BrA206	suicide	somatomotor cortex	4
4	Y440	suicide	occipital cortex	12
	Y482	suicide	occipital cortex	6
	Y558	suicide	occipital cortex	4
5	Y331	suicide	cerebellar cortex	12
	Y724	suicide	cerebellar cortex	6
	Y292	suicide	cerebellar cortex	4
6	Y591	suicide	occipital cortex	12
	Y520	suicide	occipital cortex	6
	Y787	suicide	occipital cortex	4
7	Y421	suicide	cerebellar cortex	12
	Y516	suicide	cerebellar cortex	6
	Y532	suicide	cerebellar cortex	4
8	BIB82	suicide	occipital cortex	12
	Y988	suicide	occipital cortex	6
	BI370	suicide	occipital cortex	4
9	BI269	suicide	occipital cortex	12
	Y426	suicide	occipital cortex	6
10	Y803	control	cerebellar cortex	12
	A438	control	cerebellar cortex	6
	Y880	control	cerebellar cortex	4
11	YA243	control	cerebellar cortex	12
	Y915	control	cerebellar cortex	6
	Y967	control	cerebellar cortex	4
12	Y64	control	cerebellar cortex	12
	Y726	control	cerebellar cortex	6
	B168	control	cerebellar cortex	4
13	Y872	control	cerebellar cortex	12
	Br 969	control	somatomotor cortex	4
	Br708	control	somatomotor cortex	6
14	YA 205	control	occipital cortex	6
	YB479	control	occipital cortex	12
	YB330	control	occipital cortex	4
15	Y928	control	occipital cortex	12
	Y974	control	occipital cortex	6
	Y78	control	occipital cortex	4
16	BI919	control	occipital cortex	12
	Y640	control	occipital cortex	6
	YA762	control	occipital cortex	4
17	YA626	control	occipital cortex	12
	YA29	control	occipital cortex	6
Altogether 17 pooled libraries were run; three (or two, in two cases: “run 9” and “run 17”) barcoded libraries were sequenced in a HiSeq 2000 lane. The type of the samples (suicide or control), as well as their origin (brain tissue region) were the same in the pools, with the exception of “run 13”, in which a sample from cerebellar cortex was mixed with two samples from somatomotor cortex region. The following SureSelectXT 6-bp indexing primers were used: index 4 - TGACCA; index 6 – GCCAAT; index 12 – CTTGTA.				

**Table 3:** Summary table of the quantitative data of sample pools for multiplexed sequencing.

RUN	SAMPLE	C (nM)^A^	Volume (uL)^B^	C (pg/uL)^C^	C (ng/uL)^D^	C (pg/uL)^E^	C (ng/uL)^F^	Library size^G^	Average size^H^
1	Y166	199.2	1.51	41654.8	6.4	68000	8.4	343	356
	Y919	124.8	2.40	26722		26300		358	
	Y645	74.5	4.03	16024		21500		366	
2	Y375	58.781	5.10	12521.73	6.4	8610	4.7	357	350
	Y316	57.027	5.26	11749.08		7200		338	
	Y393	86.632	3.46	18817.57		17100		356	
3	Br857	111.258	2.70	24428.34	6.4	30700	6.7	375	369
	Br333	54.035	5.55	11475.42		13100		375	
	BrA206	131.504	2.28	27907.52		19500		357	
4	Y440	39.245	7.64	8191.29	6.4	13300	7.8	352	363
	Y482	25.05	11.98	5482.86		5100		375	
	Y558	75.605	3.97	16207.71		18000		363	
5	Y331	61.145	4.91	12795.49	6.4	13500	7.2	344	357
	Y724	60.31	4.97	12715.34		20500		362	
	Y292	147.258	2.04	31909.64		23500		365	
6	Y591	103.25	2.91	22871.87	6.6	19500	7.3	376	374
	Y520	60.856	4.93	12793.51		17400		357	
	Y787	110.566	2.71	25104.72		28100		388	
7	Y421	75.488	3.97	16289.39	6.5	14200	5.7	355	353
	Y516	61.276	4.90	13104.89		11000		351	
	Y532	69.348	4.33	14934.17		14300		352	
8	BIB82	139.113	2.16	29501.49	6.0	27400	5.7	350	330
	Y988	68.642	4.37	13380.9		12600		315	
	BI370	43.857	6.84	8660.63		8200		325	
9	BI269	81.736	5.51	15828.78	6.0	11800	4.4	313	329
	Y426	54.168	8.31	11230		8210		345	
10	Y803	43.374	6.92	8970.21	6.4	6420	4.8	341	358
	A438	135.98	2.21	30004		22100		373	
	Y880	115.546	2.60	24433.7		19200		359	
11	YA243	49.12	6.11	10428.1	6.4	9450	5.7	371	361
	Y915	48.499	6.19	10605		8720		363	
	Y967	123.886	2.42	26052		25000		349	
12	Y64	139.449	2.15	29738.07	6.4	30500	7.1	357	357
	Y726	112.212	2.67	23744		28300		357	
	B168	135.267	2.22	29033.01		32100		357	
13	Y872	149.778	2.00	31377.06	6.4	28100	6.5	350	361
	Br708	116.782	2.57	24462.06		33500		355	
	Br969	100.18	2.99	21967.36		17400		377	
14	YB479	127.498	2.35	27282.84	6.3	25500	6.1	356	350
	YA205	61.118	4.91	12889.7		10030		354	
	YB330	95.388	3.15	19830.54		23900		340	
15	Y928	67.261	4.46	14431.43	6.4	14700	6.9	358	356
	Y974	99.194	3.02	21249.16		24900		367	
	Y78	90.024	3.33	18648		19800		343	
16	BI919	113.104	2.65	24609.88	6.4	24100	7.5	377	360
	Y640	161.115	1.86	34615.56		48300		369	
	YA762	119.56	2.51	24368.42		28200		335	
17	YA626	94.57	4.76	20189.16	6.5	22400	6.6	352	360
	YA29	136.8	3.29	29716.84		28000		368	
The final concentration was 30 nM in each pool. A: initial concentration of the libraries; B: volume to use in the 30 nM final pool; C: final concentration of the libraries based on Bioanalyzer (BA) measurements; D: final concentration of the pooled libraries determined by BA; E: final concentration of the single libraries measured by Qubit; F: final concentration of the libraries based on Qubit measurement; G: The length of the single libraries were determined by BA; H: The average length of the mixed pools.									

**Table 4:** Summary of the technical replicates.

PATIENT			BRAIN REGION
#194	control	occipital cortex
		occipital cortex
#230	control	somatomotor cortex
		occipital cortex
#148	suicide	occipital cortex
		occipital cortex
#143	suicide	occipital cortex
		occipital cortex
#158	suicide	cerebellar cortex
		occipital cortex
Different brain tissue samples (originating from the same region, but from different tissue pieces or different regions) from five patients (3 suicide victims and 2 controls) were used as technical parallels for sequencing.		

**Table 5:** Quality control data and statistics from whole-exome sequencing.

Sample ID	no. reads	mapped reads (%)	duplicates (%)	statistics of the analyzed region (Sure Select V5 All Exon Plus UTR, 15 bp added)											
				>Q30 reads (%)	average insert size	insert size StdDev	average depth	>5x cov (%)	>10x cov (%)	>20x cov (%)	>40x cov (%)				
A438	138452577	99.92%	5.26%	92.01%	190.8	67.7	119.9	99.60%	99.20%	97.60%	90.60%
B168	140611220	99.94%	2.28%	92.93%	184.2	55.3	134.5	99.30%	98.60%	96.40%	88.80%
BI269*	187542878	99.96%	9.18%	0.926137	157.93	45.71	168.597	99.10%	98.40%	96.40%	90.50%
BI370*	138284674	99.96%	8.02%	0.925547	159.99	48.74	125.946	98.90%	97.80%	94.80%	85.50%
BI919*	97057952	99.94%	3.96%	0.901908	183.16	63.17	88.512	99.10%	97.70%	93.30%	78.00%
BIB82	130560882	99.94%	3.30%	92.36%	180.8	57.1	122.1	99.50%	99.00%	97.20%	89.90%
Br333	93171478	99.95%	6.63%	93.12%	190.6	68.6	80.7	99.00%	97.70%	93.10%	76.40%
Br708	178033001	99.94%	3.25%	93.01%	177.5	54.2	166.6	99.50%	99.10%	98.00%	94.20%
Br857	144136294	99.96%	3.43%	92.79%	186.4	64.2	133.5	99.40%	98.90%	97.20%	90.80%
Br969	106686550	99.92%	5.23%	91.83%	185.9	68.0	95.7	99.30%	98.60%	95.90%	84.80%
BrA206	80177041	99.96%	3.44%	93.32%	185.3	62.6	72.9	98.80%	97.20%	91.40%	71.70%
Y166	76810758	99.96%	4.51%	93.30%	174.9	52.4	69.8	99.10%	97.60%	91.80%	71.30%
Y292	95054488	99.81%	2.32%	92.12%	180.9	57.4	88.5	99.20%	98.10%	94.30%	80.00%
Y316	87607856	99.95%	9.49%	92.09%	174.1	56.7	74.9	99.20%	98.20%	93.90%	76.60%
Y331	96374496	99.82%	7.61%	90.97%	179.4	57.5	85.0	99.30%	98.40%	94.80%	80.30%
Y375	99837105	99.93%	7.35%	91.82%	181.6	63.8	86.9	99.40%	98.60%	95.60%	82.20%
Y393	126108409	99.94%	6.30%	93.40%	189.4	59.8	110.9	99.60%	99.00%	97.00%	88.10%
Y421	100073380	99.93%	5.74%	92.01%	186.1	58.5	89.2	99.20%	98.30%	94.80%	81.10%
Y426	165152957	99.94%	24.86%	90.08%	175.2	60.6	113.5	99.60%	99.20%	97.90%	91.50%
Y440*	120135303	99.78%	13.84%	0.900957	177.41	62.88	97.494	99.40%	98.80%	96.30%	85.80%
Y482	83982287	99.75%	21.33%	90.80%	188.2	70.9	59.7	99.10%	97.60%	91.10%	67.20%
Y516	105766222	99.92%	6.94%	92.24%	183.6	56.0	93.3	98.80%	97.50%	93.40%	79.60%
Y520	116261151	99.80%	5.07%	91.62%	175.6	58.4	106.2	99.20%	98.30%	95.50%	85.10%
Y532	104607488	99.92%	5.72%	91.77%	185.9	56.8	93.6	99.40%	98.60%	95.50%	82.80%
Y558	99200163	99.79%	4.78%	91.02%	180.2	60.6	89.0	99.00%	97.70%	93.70%	79.80%
Y591	81128182	99.79%	3.15%	91.46%	189.9	67.4	74.3	99.20%	98.00%	93.10%	74.50%
Y64	133401783	99.93%	2.80%	92.41%	180.0	58.0	127.8	99.40%	98.80%	96.60%	88.50%
Y640	129387141	99.94%	2.19%	89.53%	176.1	55.8	125.0	99.40%	98.70%	96.40%	88.10%
Y645	114470684	99.95%	9.44%	93.06%	180.0	61.7	96.1	99.50%	98.80%	96.30%	85.30%
Y724	124302299	99.84%	5.34%	92.01%	176.0	59.8	114.0	99.30%	98.60%	96.30%	87.60%
Y726	101306831	99.93%	3.54%	91.98%	177.7	60.4	94.1	99.30%	98.40%	95.20%	82.90%
Y78	89614381	99.94%	5.49%	90.99%	176.9	56.5	79.4	98.90%	97.40%	92.10%	74.20%
Y787	114795165	99.80%	3.63%	92.68%	197.0	71.2	103.7	99.50%	98.90%	96.70%	87.20%
Y803	120331211	99.93%	12.18%	91.54%	176.2	58.9	100.5	99.40%	98.70%	96.20%	86.00%
Y872	141008850	99.93%	2.49%	92.74%	175.4	52.1	137.4	99.30%	98.60%	96.50%	89.10%
Y880	110514415	99.90%	4.95%	92.46%	181.6	65.7	85.9	99.20%	98.10%	93.90%	78.60%
Y915	101630477	99.88%	12.02%	93.19%	192.2	65.7	82.7	99.30%	98.40%	94.80%	79.80%
Y919	88647901	99.96%	3.69%	93.03%	179.2	57.4	80.1	99.30%	98.20%	93.90%	77.50%
Y928	94852671	99.94%	4.20%	91.06%	182.6	61.4	86.2	98.90%	97.40%	92.60%	76.90%
Y967	117471985	99.93%	3.69%	93.49%	179.6	58.5	108.5	99.50%	98.80%	96.40%	86.80%
Y974	119874575	99.94%	4.46%	89.84%	178.6	61.7	109.1	99.60%	98.90%	96.70%	87.50%
Y988	127546449	99.96%	5.33%	92.30%	157.7	46.3	120.2	98.70%	97.50%	93.90%	83.30%
YA205	96160167	99.95%	7.07%	90.93%	177.5	61.1	84.7	99.20%	98.10%	93.80%	78.30%
YA243	129654716	99.92%	9.35%	93.46%	186.4	69.1	110.0	99.10%	98.10%	94.80%	83.50%
YA29	170816106	99.91%	2.96%	83.51%	182.1	55.8	161.4	99.30%	98.70%	97.20%	92.20%
YA626	195722635	99.91%	6.58%	84.44%	179.3	49.7	175.8	99.60%	99.10%	97.90%	93.90%
YA762*	107955782	99.95%	4.02%	0.89896	168.69	49.99	101.205	99.20%	98.30%	95.30%	84.20%
YB330	106184066	99.94%	4.54%	91.56%	178.1	51.5	98.6	99.00%	98.00%	94.70%	82.90%
YB479	105205966	99.94%	2.95%	90.97%	181.0	56.4	98.6	99.40%	98.50%	95.30%	83.10%
The sample ID of the technical replicates are marked with asterisk.											
